# Transforming tallness: how sex steroids influence final height in Marfan syndrome

**DOI:** 10.1016/j.jped.2025.01.007

**Published:** 2025-03-13

**Authors:** Adriana Banhos Carneiro Yamaguchi, Adriana Aparecida Siviero-Miachon, Paola Matiko Martins Okuda, Lívia Cristina Oliveira e Silva, Talita de Faria Bustamante, Angela Maria Spinola-Castro

**Affiliations:** a Universidade Federal de São Paulo (UNIFESP), Escola Paulista de Medicina (EPM), Setor de Endocrinologia Pediátrica, São Paulo, SP, Brazil; b Hospital do Grupo de Apoio ao Adolescente e à Criança com Câncer (GRAACC), São Paulo, SP, Brazil; c Universidade Federal de São Paulo (UNIFESP), Departamento de Psiquiatria, São Paulo, SP, Brazil

**Keywords:** Marfan syndrome, Estradiol, Testosterone, Growth, Stature

## Abstract

**Objective:**

To evaluate the effects of hormonal treatment with sex steroids on the final height of patients with tall stature, including those diagnosed with Marfan Syndrome (MS), over 15 years in an outpatient setting.

**Methods:**

This retrospective cohort study reviewed the medical records of patients referred for tall stature. Descriptive statistics characterized the samples, while independent and paired *t*-tests assessed changes in final height (FH) and height at the start of treatment (HTS). One-way analysis of variance (ANOVA) evaluated the impact of chronological age at the initiation of therapy, bone age at the start of treatment, and pubertal stage on FH and HTS.

**Results:**

A total of 55 individuals with tall stature (51 % male) were included, among whom 35 (64 %) had clinically confirmed MS. Of these, 34 (62 %) received low-dose steroid treatment. Patients treated during pre-puberty exhibited an average height increase of 25.56 cm (95 %CI 20.40–30.73; *p* < 0.001; d = 2.86), while those treated during puberty showed an average gain of 11.93 cm (95 %CI 8.69–15.18; *p* < 0.001; d = 1.72). Early treatment before the age of 10 resulted in height gains of 13.92 cm (95 %CI 4.90–22.93; *p* = 0.006; d = 1.82) with estrogen and 6.8 cm (95 %CI 1.71–11.88; *p* = 0.010; d = 0.73) with testosterone.

**Conclusions:**

Early intervention with low doses of steroids significantly reduced final height in individuals with tall stature, including those with MS, while also minimizing dose-dependent adverse effects.

## Introduction

Tall individuals are usually referred to endocrinologists to exclude hormonal disorders leading to abnormal growth, even though some require a broad clinical investigation to be diagnosed. It is necessary to be familiar with many rare overgrowth syndromes, especially because some may have severe complications, such as Marfan syndrome (MS).^1^

MS is a genetic disorder first identified in 1896 by the French pediatrician Antoine Bernard Marfan. It shows significant phenotypic variability, with mild-to-severe and potentially fatal cases.^2^ The estimated prevalence of this syndrome is 2–3 cases per 10,000 people; however, many cases remain undiagnosed. Mutations in the FBN1 gene, located on chromosome 15q21.1, cause the condition by affecting fibrillin-1, a key component of microfibrils in connective tissue. This glycoprotein is found in the middle layer of arteries, ligaments, lungs, eye structures, and the dura mater. As a result, the cardiovascular, ocular, and skeletal systems are the most affected.^3^

Although cardiovascular factors are commonly linked to morbidity and mortality, excessive height is the primary reason for referral to specialists, as it significantly affects patients' physical and psychological well-being.^4^ Additionally, an overgrowth pattern can worsen aortic dilation and is strongly associated with a high prevalence of scoliosis.^5^

Few studies have examined the treatment or management of excessive height in patients with tall stature, including MS, and this topic remains controversial. Most discussions focus on surgical interventions, such as percutaneous epiphysiodesis or the use of supraphysiological doses of sex steroids; off-label treatment is also used for individuals with constitutional tall stature.^6^

This retrospective study evaluated the impact of sex steroid treatment on final height and height gain in patients with tall stature, including MS, over 15 years in an outpatient setting.

## Material and methods

This retrospective study was conducted at the Pediatric Endocrinology Unit of the Federal University of Sao Paulo (UNIFESP/EPM) after being approved by the Research Ethics Committee under project number 8044/2021.

### Study population and sample

The study included 55 participants with tall stature (defined as height >2.0 standard deviations, SD above the mean for age and sex),^7^ of whom 35 (64 %) were clinically diagnosed with MS based on Ghent II criteria.^8^

Initially, 97 patients were selected; however, the final cohort for the primary analysis comprised 55 patients. Approximately ten patients lacked a confirmed tall stature diagnosis, 12 did not reach the final height (FH) (defined as a growth rate of <2 cm/ year or bone age, as estimated by the Greulich-Pyle (GP) method,^9^ at least 14 years for girls and 16 years for boys), 11 used medications intermittently, and nine discontinued follow-ups.

The patients were those diagnosed with tall stature, having reached FH, assessed for bone age via the GP method,^9^ and with at least one clinical or anthropometric data point. Exclusion criteria were incomplete medical records (lacking clinical or anthropometric data), concurrent endocrine disorders, and prior growth-altering therapies unrelated to MS.

### Procedures

Clinical data from 2004 to 2021 were obtained from the medical records of patients referred to the outpatient clinic for assessment of tall stature. Key variables included:

• Clinical features: if MS diagnosis (yes or no), Tanner pubertal stage (prepubertal or pubertal),^10^^,^^11^ chronological age at the start of treatment (months), and bone age at the start of treatment (months);
• Anthropometric measures: Height at the start of therapy (HST) and FH, all in cm and Z-scores;
• Treatment characteristics: treated and untreated, and if treated: type of sex steroid (estradiol, testosterone, or combined), dosage, and treatment duration (months).

The untreated group (*n* = 21) did not receive sex steroids due to advanced bone age at the first consultation [greater than 13 years in girls (*n* = 7) and 15 years in boys (*n* = 6)]; tall stature complaints but with a height Z-score of < 2.0 (*n* = 5); or loss of follow-up during the appropriate treatment period (*n* = 3).

To standardize sex steroid use in this study, dosages were converted as follows: females were placed on estrogens, and boys were treated with testosterone alone or combined with estrogens to expedite epiphyseal closure because of poor FH prognosis. The total sex steroid dose was the average given during treatment, excluding the non-compliance periods. For boys, the monthly testosterone dose (mg) was recorded, consisting of either testosterone cypionate 200 mg (Depo-Testosterone) or a combination of testosterone salts (propionate 30 mg, phenylpropionate 60 mg, isocaproate 60 mg, and decanoate 100 mg). For patients taking conjugated estrogens (girls or boys), the dose was expressed as the equivalent daily dose of estradiol valerate (0.625 mg of conjugated estrogens equals 1 mg of estradiol valerate). For female participants, progesterone was added if vaginal bleeding occurred or after two years of starting estrogen therapy (whichever came first).^12^

### Outcome measures

FH and height gain during treatment (HGT), calculated by subtracting the HST from the FH in cm and Z-scores.

### Data analysis

A missing data analysis was conducted to ensure the integrity and reliability of the results. Little's MCAR Test (X^2^_(2374_) = 17,287, *p* = 1000) indicated that the missing values were random and substitutable. The expected maximization method estimates missing values based on participants' responses, avoids sample mean use, reduces bias, and enhances robustness. Consequently, all participants had complete data for all the variables analyzed.

Descriptive statistics were used to characterize the sample and report the continuous variables' mean and standard deviation (SD). Sample characteristics were analyzed by diagnosis (MS: yes or no), Tanner pubertal stage (pre-pubertal or pubertal), sex (female or male), and treatment type (estrogen, testosterone, or combined), and visualized using boxplots. The total dose data showed skewness; however, the parametric analysis was justified based on the following:

1. Robustness of parametric tests: Parametric tests (e.g., *t*-tests, ANOVA) were robust to moderate normality violations, particularly with 55 participants.^13^
2. Central Limit Theorem: A large sample size ensures that the sampling distribution of the mean approaches’ normality even with skewed raw data.^14^
3. Clinical relevance: The total dose variable could not be transformed because of its clinical importance.
4. Methodological consistency: Consistency was maintained across all continuous variables, including the normally distributed final height in cm and Z-score.

For a sample size of 55, approximate normality was assumed for the height Z-score data, FH (cm), and HST (cm). Groups were split into treated and untreated. Independent and paired *t*-tests checked changes in FH and HST by chronological age (up to 120 months or over 121 months) and Tanner stage (pre-pubertal or pubertal). One-way ANOVA was used to assess the impact of chronological age, Tanner stage, and bone age at the start of treatment on FH, and HGT. Analyses were performed using IBM SPSS Statistics version 30 with two-sided tests, and data are presented as mean ± SD, significance at *p* < 0.05, 95 % confidence intervals, and effect size (Cohen's d and other relevant measures).

## Results

The study included a sample of 55 tall individuals of both sexes (51 % male), of whom 35/55 patients (64 %) had a confirmed clinical diagnosis of MS. Table 1 presents the main characteristics of the MS samples.

**Table 1:** Descriptive statistics of leading MS characteristics of the sample.

Sex	MS clinical diagnosis	Total	Lens dislocation	Aortic dissection or dilation (z-score ≥ 2)	Positive family history	Systemic score ≥ 7 points
		*n*	*n*	*n*	*n*	*n*
Female	Yes	16	11	13	12	3
	No	11	2	2	6	1
Male	Yes	19	12	18	10	9
	No	9	3	2	0	1

Regarding treatment, 34/55 patients (61.8 %) (17 boys and 17 girls) received sex steroids to lower their FH, including testosterone or estrogen. Ten boys were administered both testosterone and estrogen due to inadequate FH.

Twenty-one patients out of 55 (38.2 %) (11 boys and 10 girls) did not receive any treatment, and 7 (3 boys and four girls) did not fully meet the Ghent II diagnostic criteria.

Four distinct treatment groups were described considering sex and therapy type: girls treated exclusively with oral estrogen (GTE) (*n* = 16, 29.1 %), boys treated exclusively with intramuscular testosterone (GTT) (*n* = 8, 14.5 %), boys treated with sequential therapy starting with testosterone followed by estrogen (GTET) (*n* = 10, 18.2 %), and untreated group (GNT) despite sex (*n* = 21, 38.2 %).

Among the 34 patients treated, 14/34 (41.2 %) were Tanner 1 at the beginning of treatment. Table 2 details the sample distribution by diagnosis, Tanner stage, sex, and treatment group.

**Table 2:** Sample distribution was based on diagnosis, Tanner stage, sex, and treatment group.

	Marfan Syndrome				
	No		Yes		
	Female	Male	Female	Male	Tanner at treatment
	*n*	*n*	*n*	*n*	*N*
Treatment exclusively with estrogen (GTE)	7	0	9	0	Tanner 1: 14 Tanner 2: 6 Tanner 3: 6 Tanner 4: 5 Tanner 5: 3
Treatment exclusively with testosterone (GTT)	0	0	0	8	
Treatment with both testosterone and estrogen (GTET)	0	6	0	4	
No treatment (GNT)	4	3	7	7	–

The GTE received a daily dose of 1.7 mg of estradiol valerate, the GTT received a monthly dose of 214 mg of testosterone, and the GTET received 228 mg of testosterone monthly along with a daily dose of 1.5 mg of estradiol valerate. No adverse events due to sex steroid therapy were reported. Table 3 shows the details of sex steroid treatment for each treatment group.

**Table 3:** Details of treatment with sex steroids and changes in height Z-scores.

Group (n)	Height Z score at first consultation ± SD	Chronological age at the start of treatment (years ± SD)	Bone age at the start of treatment (years ± SD)	Height Z score at the start of treatment ± SD	Treatment duration (months ± SD)	Mean dose of sex steroid (mg/day or mg/month)	Final height Z score ± SD
GTE (*n* = 16)	2.39 ± 1.09	10.58 ± 2.30	11 ± 2.75	2.62 ± 1.11	26 ± 14	1.70	1.87 ± 0.68
GTT (*n* = 8)	2.86 ± 0.95	12 ± 1.80	12.92 ± 3.90	2.74 ± 0.93	30 ± 20	214	1.88 ± 1.17
GTET (*n* = 10)	2.98 ± 1.09	*T* = 12 ± 2.5 *E* = 13.08 ± 2.60	*T* = 12 ± 2.50 *E* = 13.58 ± 1.20	*T* = 3.17 ± 1.25 *E* = 3.71 ± 1.52	*T* = 32 ± 13 *E* = 20 ± 15	*T* = 228 *E* = 1.5	2.76 ± 1.00
GNT (*n* = 21)	1.94 ± 1.12	–	–	–	–	–	1.56 ± 1.13

GTE, girls treated solely with oral estrogen; GTT, boys treated exclusively with intramuscular testosterone; GTET, boys treated with sequential therapy starting with testosterone followed by estrogen; GNT, untreated group. Data in mean ± SD.

**Figure 1:**
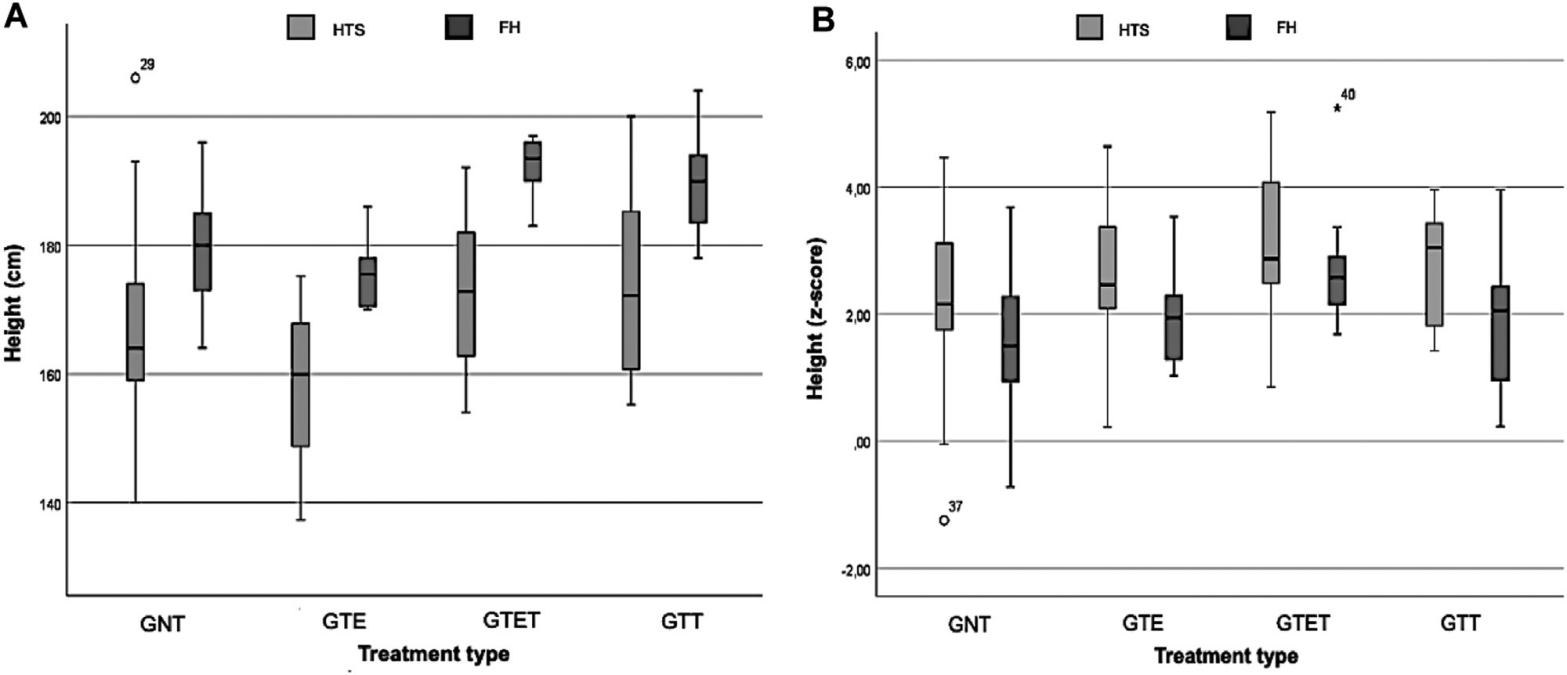
A and B. Boxplot of height evolution according to treatment. GNT, untreated group; GTE, treated with estrogen; GTET, treated with estrogen and testosterone; GTT, treated with testosterone.

The boxplot graphs (Figure 1A and B) visually analyze FH and HTS in cm and Z-scores for different treatment groups. The GNT had a mean HST of 168 cm (SD = 15.5; 95 %CI = 161.1 – 175.2) and 2.21 z-score (SD = 1.35; 95 %CI = 1.59 – 2.82); GTE had 158.2 cm (SD = 11.99; 95 %CI = 151.8 – 164.59) and 2.62 z-score (SD = 1.11; 95 %CI = 2.02 – 3.21); GTET had 174.4 cm (SD = 13.30; 95 %CI = 162.85 – 181.89) and 3.17 z-score (SD = 1.25; 95 %CI = 2.27 – 4.02); and the GTT had 173.9 (SD = 15.61; 95 %CI= 160.89 – 186.99) and 2.74 z-score (SD = 0.93; 95 %CI= 1.96 – 3.53).

Regarding FH, the GNT had 179.3 cm (SD = 9.17; 95 %CI = 175.11 – 183.46) and 1.56 z-score (SD = 1.13; 95 %CI = 1.05 – 2.07); GTE had 175.1 (SD = 4.64; 95 %CI = 172.59 – 177.53) and 1.87 z-score (SD = 0.68; 95 %CI = 1.50 – 2.23), GTET had 192.5 (SD = 4.35; 95 %CI = 189.39 – 195.61) and 2.76 z-score (SD = 1.00; 95 %CI = 2.04 – 3.48); and GTT had 189.6 cm (SD = 8.28; 182.70 – 196.55) and 1.88 z-score (SD = 1.17; 95 %CI = 0.90 – 2.86).

Figure 1A and 1B indicate that treatments, especially in the GTET and GTT groups, reduced the gap between FH and HST during the evaluated period. In the untreated group (GNT), there was more variation and heterogeneity, showing less controlled growth.

There was no effect of treatment duration or sex steroid dosage on FH among different treated groups.

In prepubertal individuals, there was a significant difference in HST and FH with a mean of 25.6 cm (SD = 8.94; 95 %CI 20.40 - 30.73; *p* < 0.001, Cohen's d = 2.86) and a z-score of −0.91 (SD = 1.02; 95 %CI −1.50 - −0.32; *p* = 0.005, Cohen's d = −0.89). In pubertal subjects, the mean difference was 11.9 cm (SD = 6.93; 95 %CI 8.69 - 15.18; *p* < 0.001, Cohen's d = 1.72) and a z-score of −0.51 (SD = 0.82; 95 %CI −0.90 - −0.12; *p* = 0.012, Cohen's d = 0.62).

Among prepubertal patients, those who started estrogen treatment before 120 months (10 years) had a mean FH difference of −13.9 cm (95 % CI: −22.93 to −4.90; *p* = 0.004; Cohen's d = 1.82). Similarly, patients treated with testosterone before 10 years had a mean FH difference of −10.5 cm (95 % CI: −17.50 to −3.59; *p* = 0.008; Cohen's d = 1.18).

There was no significant difference in FH and HGT (cm and z-score) based on bone age at treatment start in prepubertal patients treated up to 120 months (*p*-values ranged from 0.188 to 0.913).

## Discussion

In this retrospective study, the authors aimed to evaluate the impact of sex steroids on FH in tall individuals using real-world data, starting treatment upon referral to a pediatric endocrine clinic. Referrals were based on the clinical judgment of the primary care physicians.

In contemporary times, studying tall children or teenagers is challenging. Height is often seen positively, so fewer parents seek medical advice for taller kids. Consequently, syndromes affecting height may be undiagnosed until other health issues, like scoliosis or aortic dilation, emerge.^1^

It is essential to clarify that the primary purpose of starting treatment for all male or female patients was height prognosis (> 2.0 SD), which was also the main reason they were referred to the endocrine clinic. The treatment for patients diagnosed with MS is straightforward. Those not meeting the full clinical criteria were also treated for similarly poor height prognosis. While all had some critical MS features, without molecular confirmation, the authors cannot rule out MS. Patients with MS experience delayed growth plate closure due to fibrillin disorder, which extends their growth period.^2^

This study revealed significant differences in the growth patterns and FH outcomes between treated and untreated groups, especially in the GTET and GTT groups. Interestingly, these effects did not correlate with treatment duration or sex steroid dosage, suggesting complex growth regulation mechanisms.

Studies on high-dose estrogen for treating MS and tall stature are limited. Estrogen types and doses vary from 50 to 300 mg/day of ethinyl estradiol (5–30 mg/day of estradiol valerate). It remains the most common treatment for controlling tall stature.^15^

Table 1 located in the supplementary material, provides a summary of key studies on the treatment of tall stature in patients with MS. Given the rarity of the syndrome, all studies were conducted with small sample sizes.^4^^,^^5^^,^^16^, ^17^, ^18^

In this study, the average estrogen dose was 1.5–1.7 mg/day of estradiol valerate, lower than the average reported in the literature. Lower estrogen doses were used because of uncertainty concerning long-term adverse effects and the safety of high doses of sex steroids.^19^

Initiating sex steroid therapy at a lower age in prepubertal tall patients (< 10 years) had the most positive effect on FH. Surprisingly, bone age at the beginning of sex steroid therapy did not affect FH.^4^

By the present data, Lee et al. also found a significant reduction in FH in girls with MS who began treatment before 11 years of age.^5^ Similarly, Kim et al. demonstrated that hormonal therapy with estradiol valerate, when initiated before 10.5 years of age, effectively reduced the final height by approximately 10 cm in girls with MS.^18^ Nonetheless, both studies used high doses of sex steroids.

This study is the first to utilize combined testosterone and estrogen treatment in a subgroup of male patients with a poorer prognosis (GTET), acknowledging the significance of estrogen in epiphyseal closure.^6^^,^^20^ Although the GTET group achieved a FH of 2.76 z-score, their height decreased from the initiation of therapy.

The retrospective nature of this study had several limitations. The severity of scoliosis, assessed using Cobb angles, was not evaluated, and no adjustments were made for the correction formulas suggested by some authors. Children with scoliosis were included in the height analysis without accounting for potential biases from conditions such as kyphosis or lower limb length discrepancies, which can affect stature.^21^^,^^22^ In addition, multiple professionals conducted bone age assessments, increasing the risk of inter-observer variability.

The medication regimen lacked standardization, with variations in dose, type of sex steroids, and treatment duration of > 15 years of follow-up. A more robust study design - prospective, longitudinal, multicenter, with a larger sample and standardized drug protocols - would be ideal for assessing different sex steroid doses and long-term side effects. Such a study has yet to be published.

This retrospective study evaluated the impact of hormonal treatment with sex steroids on the FH of patients with tall stature, including those with MS, monitored over 15 years in an outpatient setting. The results showed that early intervention with low doses of steroids significantly reduced FH in tall populations, including those with MS, while also reducing dose-dependent adverse events.

## Conflicts of interest

The authors declare no conflict of interest.

## Appendix Supplementary materials

Image, application 1
