# Supplementary material for: Transforming tallness: how sex steroids influence final height in Marfan syndrome
Source: J Pediatr (Rio J). 2025 Mar 13;101(3):424–9. doi: 10.1016/j.jped.2025.01.007 (PMC12039514; doi:10.1016/j.jped.2025.01.007)
Supplement: Supplementary file 1 [file mmc1.docx]

**JPED-D-24-00523_ Supplementary material**

**Supplementary material**

**Table 4** Key studies on the treatment of tall stature in patients with MS using sex steroids.

|  | n | Duration treatment (mean) | Type and doses |
| --- | --- | --- | --- |
| Knudtzon & Aaskog (1988)^[16]^ | 5 | Two years | Estradiol valerate (n=4) 6 to 8 mg/day Ethinyl estradiol (n=1) 250 mcg/day |
| Rozendaal et al. (2005)^[4]^ | 22  21 | 2.2 years  1.7 years | Ethinyl estradiol 100 to 300 mcg/day Testosterone 100-500 mg every two weeks |
| Ucar et al. (2009)^[17]^ | 4 | Minimum 1.9 years; maximum eight years | Ethinyl estradiol 50 to 100 mcg/day |
| Lee et al. (2016)^[5]^ | 8 | Three years | Estradiol valerate 2 to >12 mg/day |
| Kim et al. (2021)^[18]^ | 17 | Minimum 2.5 years; maximum 4.6 years | Estradiol valerate 2 to 12 mg/day |
| Present study | 16  8  10 | Two years  2.5 years  1.6 years + 2.6 years | Estradiol valerate 1.7 mg/day  Testosterone 214 mg every four weeks  Estradiol valerate 1.5 mg/day + Testosterone 228 mg every 4 weeks |
